# Identification of Cytosolic DNA Sensor cGAS-STING as Immune-Related Risk Factor in Renal Carcinoma following Pan-Cancer Analysis

**DOI:** 10.1155/2022/7978042

**Published:** 2022-08-09

**Authors:** Zheng Wu, Ying Lin, Li-Min Liu, Yan-Li Hou, Wei-Ting Qin, Lei Zhang, Shu-Heng Jiang, Qin Yang, Yong-Rui Bai

**Affiliations:** ^1^Department of Radiation Oncology, Renji Hospital, School of Medicine, Shanghai Jiao Tong University, Shanghai 200127, China; ^2^Department of Medical Oncology, Fudan University Shanghai Cancer Center, Shanghai 200032, China; ^3^Department of Oral Pathology, Shanghai Ninth People's Hospital, College of Stomatology, Shanghai Jiao Tong University School of Medicine, Shanghai 200011, China; ^4^State Key Laboratory of Oncogenes and Related Genes, Shanghai Cancer Institute, Renji Hospital, School of Medicine, Shanghai Jiao Tong University, Shanghai 200240, China; ^5^Shanghai Ninth People's Hospital, Shanghai Jiao Tong University School of Medicine, Shanghai 200011, China; ^6^Shanghai Institute of Precision Medicine, Shanghai 200125, China

## Abstract

**Background:**

The cytosolic DNA sensor cyclic GMP-AMP synthase (cGAS) plays critical functions in innate immune responses via the production of the second messenger cyclic guanosine monophosphate-adenosine monophosphate (cGAMP), which stimulates the adaptor stimulator of interferon genes (STING). However, the clinical relevance and prognostic value of the cGAS-STING pathway in human cancers remains largely unexplored.

**Methods:**

A gene signature related to the cGAS-STING score was identified. The pan-cancer landscape of cGAS-STING expression was calculated using the RNAseq data acquired from the TCGA cohort. Tumor-infiltrating immune cells (TIICs) were determined by the ssGSEA method. Kaplan–Meier curves, Cox regression analyses, and the area under the curve (AUC) were employed to decipher the predictive value of cGAS-STING risk score and TIICs across several human cancers.

**Results:**

Most tumor tissues displayed a higher cGAS-STING score compared with their corresponding nontumor tissues, except for prostate adenocarcinoma (PRAD) and uterine corpus endometrial carcinoma (UCEC). Higher cGAS-STING score was closely associated with poor clinical outcome of kidney renal clear cell carcinoma (KIRC) and kidney renal papillary cell carcinoma (KIRP), whereas the cGAS-STING score predicted a better prognosis in pheochromocytoma and paraganglioma (PCPG). Enrichment analysis showed that cGAS-STING was profoundly implicated in diverse immune-related pathways in KIRC, KIRP, and PCPG. Significant positive correlations were noticed between cGAS-STING score and TIICs, including activated CD8+ T cells, activated CD4+ T cells, monocytes, and mast cells. Finally, the cGAS-STING score was revealed to be an independent prognostic factor for KIRC patients and possessed a strong predictive power for the prognostic evaluation of KIRC and KIRP patients.

**Conclusions:**

We constructed a cGAS-STING gene signature to predict survival and tumor immunity across human cancers, which can serve as a novel prognostic indicator and therapeutic target, especially in KIRC and KIRP.

## 1. Introduction

As a first line of defense against pathogen infection, the innate immune system deploys germline-encoded receptors named pattern recognition receptors (PRRs) to detect pathogen-associated molecular patterns (PAMPs) and danger damage-associated molecular patterns (DAMPs) [1, 2]. For extracellular pathogens, transmembrane receptors, including Toll like receptors (TLRs) and C-type lectin receptors (CLRs), are the main sensors [3]. Activation of these signaling cascades triggers transcription factor activation and results in the expression of multiple genes involved in immune and inflammatory responses [4]. For extranuclear DNA or extracellular RNA as DAMP signals, nucleic acid (NA) sensing is an essential mechanism of the innate immunity [5].

Recently, various classes of cytosolic NA sensors have been discovered [6, 7]. These include RNA sensors and DNA sensors. The retinoic acid-inducible gene-I- (RIG-I-) like receptor (RLR) family is responsible for cytosolic RNA sensing [8], while the cyclic guanosine monophosphate-adenosine monophosphate (cGAMP) synthase (cGAS) is the most well-known and best-studied cytosolic DNA sensor [9, 10]. cGAS can directly bind to cytosolic DNA and then catalyzes the synthesis of 2′-3′-cyclic GMP-AMP (cGAMP) from ATP and GTP [11]. cGAMP acts as a secondary messenger to bind the endoplasmic reticulum- (ER-) membrane adaptor protein stimulator of interferon genes (STING), which traffics to the ER-Golgi intermediate compartment and the Golgi apparatus [12, 13]. STING activates IKK and recruits tank-binding kinase 1 (TBK1), which in turn recruits interferon regulatory factor 3 (IRF3) for phosphorylation by TBK1 and activates nuclear factor kappa B (NF-*κ*B) [14, 15]. Finally, activation of this cascade converges on the expression of transcription of type I interferons (IFNs), interferon-stimulated genes (ISGs), and other antiviral and/or proinflammatory cytokines and chemokines [16].

Self DNA from dying or damaged cancer cells, as an important danger DAMPs signal, triggers the cGAS-STING signaling pathway to induce IFNs, which is critical for intrinsic antitumor immunity [17–20]. However, many studies also suggested that cancer cells developed strategies to suppress the cGAS-STING pathway, likely for immune evasion during tumor development and progression [17]. For example, STING-dependent DNA sensing enhances tolerogenic states and promotes tumor growth in tumors characterized by low antigenicity via activation of indoleamine 2,3 dioxygenase (IDO) [21]; Moreover, brain metastatic cancer cells can transfer cGAMP to neighboring astrocytes, resulting in activation of the STING pathway and production of inflammatory cytokines such as IFN*α* and tumor necrosis factor (TNF), which ultimately facilitate tumor metastasis [22]. However, how the cGAS-STING pathway might lead to stimulatory immune responses versus oncogenic activities in different tumor types remains largely obscure.

In the present study, we performed a pan-cancer analysis using the cGAS-STING-related risk score. Through analyzing multiple levels of data from The Cancer Genome Atlas (TCGA), we comprehensively identified the molecular features, its association with tumor-infiltrating immune cells (TIILs), and the clinical relevance of the cGAS-STING pathway across a wide variety of cancer types. The capability of the cGAS-STING score in predicting the prognosis of cancer patients was also validated. It is anticipated that the comprehensive pan-cancer analysis could guide cGAS-STING-dependent cancer therapy with better precision.

## 2. Materials and Methods

### 2.1. Data Acquisition

The Cancer Genome Atlas (TCGA) research network has profiled and analyzed a large collection of clinical and molecular data of over 10,000 tumor patients across 33 different tumor types. Transcriptome RNA-seq data of 33 cancers were extracted from the TCGA database (/https://xena.ucsc.edu/). The corresponding clinicopathological parameters including sex, age, TNM stage, lymph node metastasis, histological type, and follow-up information were also extracted for further analysis. The cancer types were shown as follows: BLCA, bladder urothelial carcinoma; BRCA, breast invasive carcinoma; CESC, cervical squamous cell carcinoma and endocervical adenocarcinoma; COAD, colon adenocarcinoma; ESCA, esophageal carcinoma; GBM, glioblastoma multiforme; HNSC, head and neck squamous cell carcinoma; KICH, kidney chromophobe; KIRC, kidney renal clear cell carcinoma; KIRP, kidney renal papillary cell carcinoma; LIHC, liver hepatocellular carcinoma; LUAD, lung adenocarcinoma; LUSC, lung squamous cell carcinoma; PAAD, pancreatic adenocarcinoma; PCPG, pheochromocytoma and paraganglioma; PRAD, prostate adenocarcinoma; READ, rectum adenocarcinoma; STAD, stomach adenocarcinoma; THCA, thyroid carcinoma; and UCEC, uterine corpus endometrial carcinoma.

### 2.2. Identification and Assessment of cGAS–STING Genes

**Figure 1:**
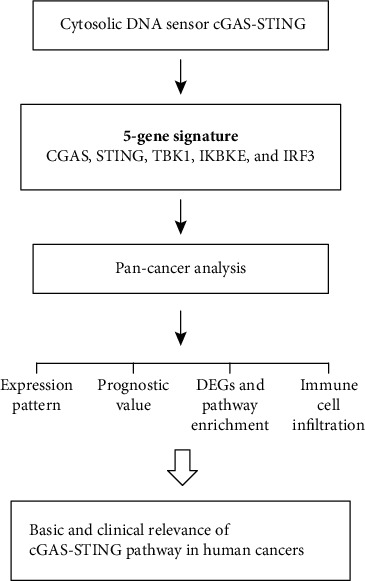
Graphic abstract for the cGAS-STING pathway and method flow.

According to previous review works [9, 10, 23], 5 genes critically involved in the cGAS-STING signaling pathway were identified, including CGAS, TMEM173 (STING), TBK1, IKBKE, and IRF3. Next, the differential expression of this gene signature was assessed in human cancers. The cGAS-STING gene signature score of the different tumor samples was determined by ssGSEA in the R Bioconductor package Gene Set Variation Analysis (GSVA, v.3.5) with default parameters (Figure 1).

### 2.3. Identification of Differentially Expressed Genes

According to the score of the cGAS-STING gene signature, the tumor samples were divided into two groups using the median score as a cutoff. Differentially expressed genes (DEGs) between the high-score and low-score subgroups were then identified using the “limma” package. Genes with *P* value < 0.05 and |log_2_FC| ≥ 1 were seemed as a DEG in each cancer type.

### 2.4. Functional Enrichment Analysis

Gene Ontology (GO) analysis was employed to annotate the DEGs related to the cGAS-STING signal. Briefly, we performed GO analysis by using the “clusterProfiler” (http://www.bioconductor.org/packages/release/bioc/html/clusterProfiler.html) R package. The results for BP (biological process), CC (cellular component), and MF (molecular function) were finally visualized. The enriched signaling pathways were then visualized with the “ggplot2” (https://cran.r-project.org/web/packages/ggplot2/index.html) R package. Two-tailed *P* less than 0.05 was considered statistically significant.

### 2.5. Identification of TIIC Subpopulations

Cell-type identification by Estimating Relative Subsets of RNA Transcripts (CIBERSORT) algorithm was utilized to calculate the immune scores for each patient. The association between the cGAS-STING score and 28 tumor-infiltrating immune cells (TIICs) was then analyzed. The immune cell types enrolled in the analysis included activated B cells, activated CD4 T cells, activated CD8 T cells, central memory CD4 T cells, central memory CD8 T cells, effector memory CD4 T cells, effector memory CD8 T cells, gamma delta T cells, immature B cell, memory B cell, regulatory T cell, T follicular helper cell, type 1 T helper cell, type 17 T helper cell, type 2 T helper cell, activated dendritic cell, CD56 bright natural killer cell, CD56dim natural killer cell, eosinophil, immature dendritic cell, macrophage, mast cell, monocyte, myeloid-derived suppressor cell (MDSC), natural killer cell, natural killer T cell, neutrophil, and plasmacytoid dendritic cell [24]. The estimated fraction of individual immune cell types was calculated using single sample gene set enrichment analysis (ssGSEA) in the R package GSVA.

### 2.6. Clinical Significance Analysis

To determine the prognostic value of the cGAS-STING score, all patients were divided into two groups based on the median expression of the cGAS-STING score. The log-rank test was used to compare the survival rate difference between the two groups. Multivariable Cox regression was utilized to test whether the cGAS-STING score was an independent risk factor among all risk factors. *P* values less than 0.05 were considered statistically significant. The survival analysis was performed using R package survival and survminer.

### 2.7. Statistical Analysis

All statistical analysis was conducted with the R software (version 3.6.1). Receiver-operating characteristic (ROC) curve analysis was carried out to test the sensitivity and specificity of the risk prediction according to the cGAS-STING score. The Kruskal-Wallis test was used to compare immune scores between two subgroups. Kaplan-Meier method was used to compare survival curves. *P* values less than 0.05 were considered statistically significant.

## 3. Results

### 3.1. Expression Landscape of the cGAS-STING Sensor across Human Cancers

**Figure 2:**
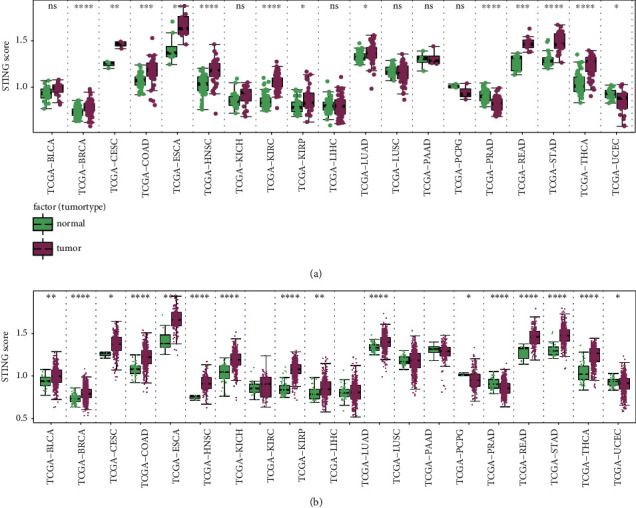
Expression landscape of the cGAS-STING sensor across human cancers. (a) The expression score of the cGAS-STING signal in paired tumor and nontumor tissues. (b) Significant difference of the cGAS-STING score between tumor and normal samples. BLCA: bladder urothelial carcinoma; BRCA: breast invasive carcinoma; CESC: cervical squamous cell carcinoma and endocervical adenocarcinoma; COAD: colon adenocarcinoma; ESCA: esophageal carcinoma; HNSC: head and neck squamous cell carcinoma; KICH: kidney chromophobe; KIRC: kidney renal clear cell carcinoma; KIRP: kidney renal papillary cell carcinoma; LIHC: liver hepatocellular carcinoma; LUAD: lung adenocarcinoma; LUSC: lung squamous cell carcinoma; PAAD: pancreatic adenocarcinoma; PCPG: pheochromocytoma and paraganglioma; PRAD: prostate adenocarcinoma; READ: rectum adenocarcinoma; STAD: stomach adenocarcinoma; THCA: thyroid carcinoma; UCEC: uterine corpus endometrial carcinoma. ^∗^*P* < 0.05; ^∗∗^*P* < 0.01; ^∗∗∗^*P* < 0.001; ^∗∗∗∗^*P* < 0.0001.

Given the fact that cGAS-STING is an important biomarker for cancer diagnosis and targeting the cGAS-STING signaling pathway represents a new therapeutic strategy, we evaluated the score of cGAS-STING signal in different tumors and their corresponding normal tissues. Data from TCGA database revealed that the cGAS-STING score was significantly higher in BRCA, CESC, COAD, ESCA, HNSC, KIRC, KIRP, LUAD, READ, STAD, and THCA tumor tissues compared to that in paired normal tissues, suggesting that cGAS-STING might play oncogenic roles in the development of those tumor types (Figure 2). In contrast, PRAD and UCEC had lower cGAS-STING scores compared with their corresponding control tissues. Notably, COAD had the highest cGAS-STING score while BRCA had the lowest among those tumor types (Figure 2(a)). Moreover, a similar phenomenon was also noticed in unpaired tumor and nontumor tissues (Figure 2(b)).

### 3.2. Prognostic Analysis of the cGAS-STING Sensor across Human Cancers

**Figure 3:**
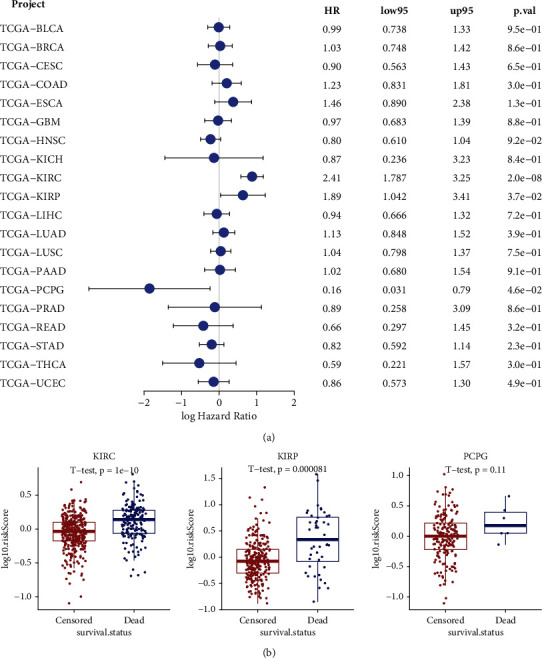
Prognostic analysis of the cGAS-STING sensor across human cancers. (a) Univariate Cox regression analysis was performed to determine the prognostic values of the cGAS-STING sensor. (b) Expression pattern of cGAS-STING in KIRC, KIRP, and PCPG with different survival status (censored or death). BLCA: bladder urothelial carcinoma; BRCA: breast invasive carcinoma; CESC: cervical squamous cell carcinoma and endocervical adenocarcinoma; COAD: colon adenocarcinoma; ESCA: esophageal carcinoma; GBM: glioblastoma multiforme; HNSC: head and neck squamous cell carcinoma; KICH: kidney chromophobe; KIRC: kidney renal clear cell carcinoma; KIRP: kidney renal papillary cell carcinoma; LIHC: liver hepatocellular carcinoma; LUAD: lung adenocarcinoma; LUSC: lung squamous cell carcinoma; PAAD: pancreatic adenocarcinoma; PCPG: pheochromocytoma and paraganglioma; PRAD: prostate adenocarcinoma; READ: rectum adenocarcinoma; STAD: stomach adenocarcinoma; THCA: thyroid carcinoma; UCEC: uterine corpus endometrial carcinoma; HR: hazard ratio. ^∗^*P* < 0.05; ^∗∗^*P* < 0.01; ^∗∗∗^*P* < 0.001; ^∗∗∗∗^*P* < 0.0001.

Next, we assessed the prognostic value of the cGAS-STING sensor for pan-cancer overall survival. Cancer cases were divided into high-expression and low-expression groups based on the score of cGAS-STING gene signature, and correlation between the level of cGAS-STING score and the prognosis of patients was investigated. As a result, 20 tumor types were available for analysis. In detail, high cGAS-STING score was linked to poor prognosis of overall survival for KIRC (HR = 2.41, 95% CI 1.878-3.25, *P* = 2.0*e* − 08) and KIRP (HR = 1.89, 95% CI 1.042-3.41, *P* = 3.7*e* − 02) within TCGA project (Figure 3(a)). Additionally, cGAS-STING score was associated with a better overall survival in PCPG (HR = 0.16, 95% CI 0.031-0.79, *P* = 4.6*e* − 02) (Figure 3(a)). Therefore, we selected KIRC, KIRP, and PCPG for further analysis. The results showed that KIRC and KIRP patients with dead survival status had higher scores of cGAS-STING signal, while no significant difference was found in PCPG patients (Figure 3(b)).

### 3.3. Alterations Related to the cGAS-STING Sensor in KIRC, KIRP, and PCPG

**Figure 4:**
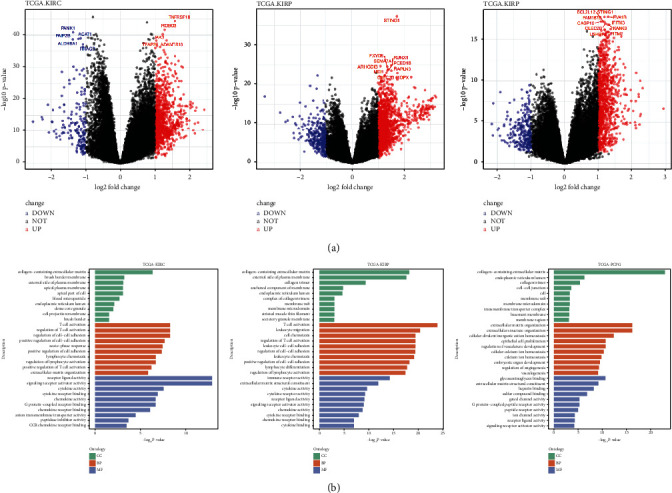
Alterations related to the cGAS-STING sensor in KIRC, KIRP, and PCPG. (a) Volcano plots displayed the differentially expressed genes between tumors with higher cGAS-STING score and tumors with the lower cGAS-STING score; the median value was set as a cutoff. (b) Gene Ontology analysis of differentially expressed genes was performed in KIRC, KIRP, and PCPG, respectively.

To elucidate the molecular implication of cGAS-STING sensor in the carcinogenesis of KIRC, KIRP, and PCPG, we first identified DEGs by using a dichotomous score of cGAS-STING. As displayed in Figure 4(a), many DEGs were revealed in the corresponding tumor types. By annotation of those DEGs with Gene Ontology (GO), many cancer-related pathways were visualized (Figure 4(b)). In KIRC, DEGs related to the cGAS-STING sensor mainly participated in cytokine binding, cytokine receptor activity, immune receptor activity, lymphocyte differentiation, leukocyte chemotaxis, leukocyte cell-cell adhesion, cell chemotaxis, etc. In KIRP, DEGs were enriched in CCR chemokine receptor binding, peptidase inhibitor activity, G-protein coupled receptor, positive regulation of T-cell activation, lymphocyte chemotaxis, acute phase response, etc. Different from KIRC and KIRP, DEGs were enriched in receptor ligand activity, ion channel activity, G-protein coupled peptide receptor activity, regulation of angiogenesis, embryonic organ development, extracellular structure organization, etc. in PCPG (Figure 4(b)). These findings prompted a link between the cGAS-STING sensor and TIICs in the tumor microenvironment.

### 3.4. Correlation between the cGAS-STING Sensor and TIICs in KIRC, KIRP, and PCPG

**Figure 5:**
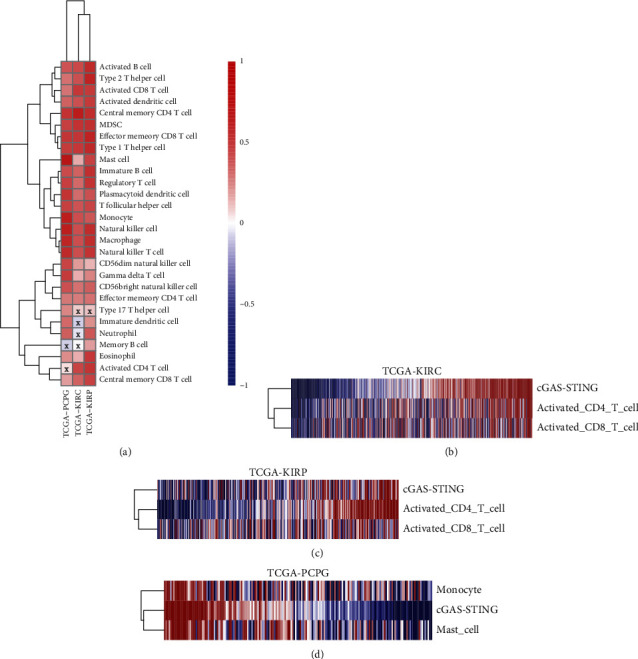
Correlation between the cGAS-STING sensor and TIICs in KIRC, KIRP, and PCPG. (a) Heatmap showed the correlation efficiency between cGAS-STING sensor and different immune cells in KIRC, KIRP, and PCPG; infiltrating immune cells in different groups were calculated by CIBERSORT. (b) Heatmap showed the activity score of cGAS-STING sensor on activated CD4 T cells and activated CD8 T cells in KIRC. (c) Heatmap showed the activity score of the cGAS-STING sensor on activated CD4 T cells and activated CD8 T cells in KIRP. (d) Heatmap showed the activity score of the cGAS-STING sensor on monocyte and mast cells in PCPG.

TIICs are an essential part of the tumor microenvironment that modulate tumor initiation and progression of many cancers [25]. The quantity and activity status of TIICs are also important predictive factors for patients' prognosis [26]. To further dissect the connection between the cGAS-STING sensor and TIICs, we interrogated 28 immune cell types for detailed analysis, which involves both adaptive immunity and innate immunity. The results showed that the cGAS-STING score was associated with infiltration of many immune cells in KIRC, KIRP, and PCPG (Figure 5(a)). In KIRC and KIRP, the cGAS-STING scores had significant correlations with activated CD4 T cells and activated CD8 T cells (Figures 5(b) and 5(c)). In contrast, the cGAS-STING score was remarkably correlated with the infiltration levels of mast cells and monocytes in PCPG (Figure 5(d)). Taken together, the results indicated that the cGAS-STING signal was tightly correlated with the extent of immune infiltration in cancers.

### 3.5. Multivariable Cox Regression Analysis of the cGAS-STING Sensor and TIICs in KIRC, KIRP, and PCPG

**Figure 6:**
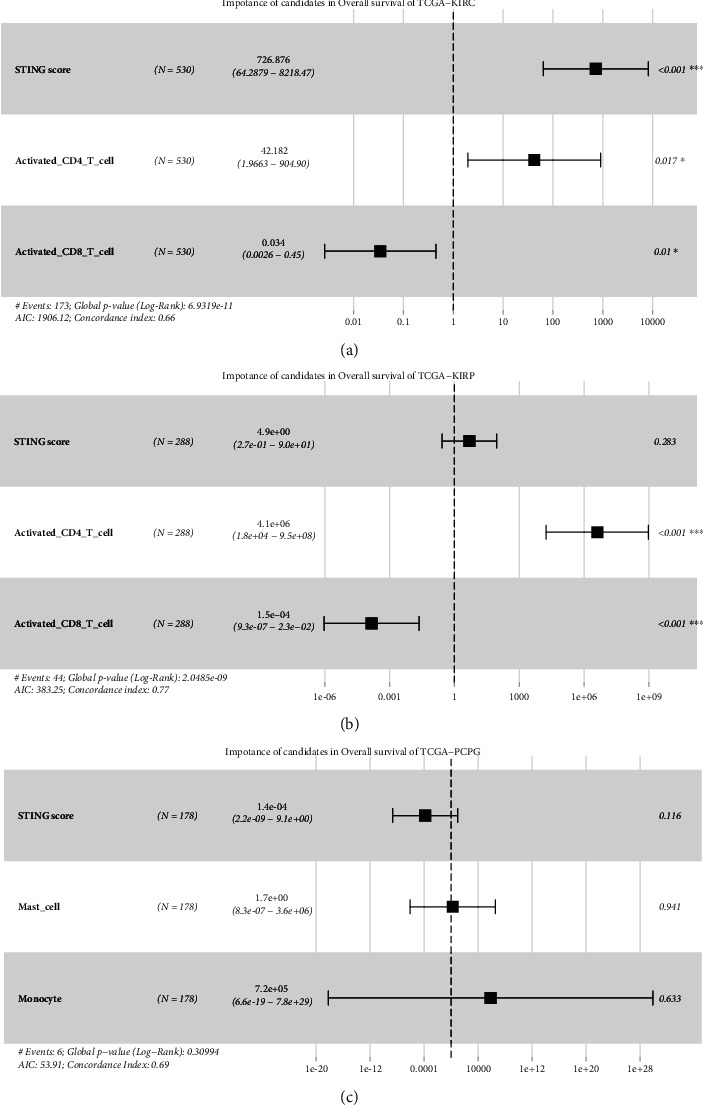
Multivariable Cox regression analysis of the cGAS-STING sensor and TIICs in KIRC, KIRP, and PCPG. (a) Multivariable Cox regression analyses of the cGAS-STING sensor, activated CD4 T cells, and activated CD8 T cells in KIRC. (b) Multivariable Cox regression analyses of the cGAS-STING sensor, activated CD4 T cells, and activated CD8 T cells in KIRP. (c) Multivariable Cox regression analyses of the cGAS-STING sensor, monocytes, and mast cells in PCPG. ^∗^*P* < 0.05; ^∗∗∗^*P* < 0.001.

To investigate whether the prognostic value of the cGAS-STING score is independent of TIICs associated with tumor progression, the multivariable Cox regression analysis was performed using the cGAS-STING score, activated CD4 T cells, activated CD8 T cells, mast cells, and monocytes as covariates. Interestingly, the cGAS-STING score was revealed as an independent risk factor for KIRC patients (HR = 726.876, 95% CI 64.29-8218.47, *P* < 0.001) (Figure 6(a)). In KIRP, activated CD4 T cells but not cGAS-STING score was identified as an independent risk factor (HR = 4.1 + *e*06, 95% CI 1.8 + *e*06 − 9.5 + *e*08, *P* < 0.001) (Figure 6(b)). In PCPG, no factors were related to a worse prognosis as a continuous variable (Figure 6(c)). Therefore, our findings suggested that the cGAS-STING score exhibited unbiased efficacy for predicting the prognosis of KIRC patients.

### 3.6. The Clinical Value of the cGAS-STING Score in KIRC, KIRP, and PCPG

**Figure 7:**
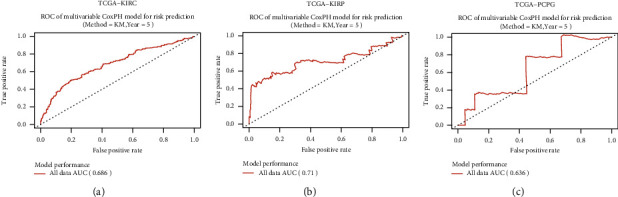
The clinical value of the cGAS-STING score in KIRC, KIRP, and PCPG. (a) Receiver-operating characteristic (ROC) analysis of the sensitivity and specificity of the risk prediction by the cGAS-STING score in KIRC. (b) ROC analysis of the sensitivity and specificity of the risk prediction by the cGAS-STING score in KIRP. (c) ROC analysis of the sensitivity and specificity of the risk prediction by the cGAS-STING score in PCPG. AUC indicates the area under the ROC curve.

To further investigate the clinical value of the cGAS-STING risk score in the management of cancer patients, receiver-operating characteristic (ROC) analysis was conducted to determine the sensitivity and specificity of survival prediction. The area under the ROC curve (AUC) was evaluated with the cGAS-STING risk score. As a result, we observed that the cGAS-STING risk score risk model possessed a strong predictive power for the prognostic evaluation of KIRC patients (AUC = 0.686) (Figure 7(a)). In KIRP, the efficacy of diagnosis using the cGAS-STING risk score was higher than KIRC, with an AUC of 0.71 (Figure 7(b)). However, the cGAS-STING risk score could not efficiently distinguish the prognosis of PCPG patients (Figure 7(c)).

## 4. Discussion

The involvement of DNA sensors in the antitumor response makes them attractive drug targets in tumor therapy, particularly for the cGAS-STING pathway and its downstream transcription factors IRF3 and NF-*κ*B [27]. Due to the important mechanism in fuelling the development of inflammation and immune response, the cGAS-STING pathway has emerged as a critical regulator of cancer development. Most studies have proven that the activation of cGAS-STING signaling stimulates antitumor immune responses. However, activation of STING can recruit not only immune-supporting cells to inhibit malignant transformation but also immunosuppressive cells to drive tumor progression [28]. In this study, we comprehensively analyzed the expression pattern, prognostic value of the cGAS-STING pathway, as well as its correlation with TIICs in the tumor microenvironment in an extensive manner.

cGAS is activated by interacting with double-stranded DNA (dsDNA). The second messenger, cGAMP, then activates STING on the ER, which forms a tetramer through high-order oligomerization and translocates from ER to ER-Golgi intermediate region. At the Golgi, palymitoylation of STING has been proposed to recruit TBK1 and IRF3, at which point IRF3 translocates to the nucleus and performs its transcriptional function in expressing immune-stimulating genes (ISG) and type 1 IFN. Meanwhile, STING also activated IKK-mediated induction of NF-*κ*B-driven inflammatory genes [29]. According to KEGG databases and related studies, using a 5-gene cGAS-STING signature, we identified the activity of the cGAS-STING pathway in many cancer types based on the data of TCGA. In contrast to the well-documented antitumor activities induced by the cGAS-STING pathway, we revealed that the majority of cancer types had higher cGAS-STING scores compared with their normal counterparts. Consistent with our findings, a previous study also reported that upregulated cGAS-STING signaling is negatively associated with the infiltration of immune cells and a high level of cGAS-STING signaling predicts a poor prognosis in certain cancers [30]. Different from that study, a gene set signature instead of a single gene to indicate the activity of the cGAS-STING pathway was adopted. In our study, patients with a high cGAS-STING score had a worse clinical outcome in KIRC and KIRP. These findings suggest a tumor-promoting effect of the cGAS-STING pathway in KIRC and KIRP. Consistent with our findings, Msaouel et al. reported that renal medullary carcinoma is characterized by high replication stress and an abundance of focal copy number alterations associated with activation of the cGAS-STING pathway [31]. Moreover, cGAS-STING-dependent DNA-sensing of micronuclei in tumor cells can couple chromosome instability to tumor metastasis [32], suggesting a contradictory role for the pathway in cancer biology. Therefore, the distinct roles of the cGAS-STING pathway might be tumor-specific, and future therapies targeting the cGAS-STING pathway should be tailored to each cancer type.

Activation of the cGAS-STING pathway can induce the recruitment of immune cells into the tumor microenvironment, such as CD8+ T cells, dendritic cells (DCs), and natural killer (NK) cells. More importantly, STING agonists can elevate immunogenicity in nonimmunogenic tumors, thus improving the efficacy of immunotherapy [33]. Accumulated studies revealed the greatest promise of application of STING agonists in combination with other immuno-oncology agents, such as the immune checkpoint inhibitors anticytotoxic T lymphocyte antigen 4 (CTLA-4), antiprogrammed cell death 1 (PD-1), and its ligand antiprogrammed cell death 1 ligand 1 (PD-L1) [34]. In this study, we also revealed a close link between the cGAS-STING pathway and TIICs in KIRC, KIRP, and PCPG. Inflammation is a common feature and is an essential factor for cancer development. Deciphering the tumor immune microenvironment reprogrammed by the cGAS-STING pathway is of great importance to understand cancer development in each cancer type. Activation of cGAS-STING signaling pathway has a bidirectional effect, which can not only activate immune-supporting cells to play an antitumor role but also create an immunosuppressive environment, thus promoting tumor formation and metastasis [28]. Therefore, more works are encouraged to uncover cGAS-driven inflammation microenvironment in different cancer types to enable therapy.

Prognosis prediction analysis is important in cancers as it can aid better clinical management of cancer patients. Emerging studies have reported a functional link between the cGAS-STING pathway and tumor diseases [19–21]. However, whether the cGAS-STING score can predict patients' prognosis remains to be answered. In this study, we for the first time analyzed the relationship between overall expression level of cGAS-STING signaling pathway and tumor prognosis. The results demonstrated that the expression level of cGAS-STING is a risk factor in KIRC and KIRP patients, and the risk score is powerful to predict patients' prognosis. We speculated that the unique characteristics of cGAS-STING genes in KIRC and KIRP may be related to the unique tumor microenvironment. Renal cancer has a high level of immune invasion [35]. More specifically, T cells, CD8+, T helper cell 1 (Th1), dendritic cells (DC), neutrophils, and cytotoxic cells scored highest in clear cell renal cell carcinoma (ccRCC), while Th2 and regulatory T cells (Treg) scores were relatively low, suggesting an overall proinflammatory effect of ccRCC [36]. T cell activation status in invasive tumors is a stronger prognostic indicator. Unlike most other cancer types, CD8+ T cell infiltrates are generally higher in ccRCC with a poor prognosis, suggesting that the invasive CD8+ T cell pool may be dominated by suppressed and dysfunctional cells [37]. Another study showed that accumulation of CD39+CD8+ T cells indicated poor prognosis in ccRCC patients [38]. Thus, a further independent validation cohort is warranted to confirm the predictive value of the cGAS-STING score and TIICs in the survival prognosis of renal cancers.

There may be several limitations in this study. Because many molecular components are included in the cGAS-STING pathway, different gene signatures related to cGAS-STING might generate minor inconsistencies for the molecular and clinical relevance of the cGAS-STING pathway. In addition, all analysis was performed by the TCGA data; further validation in the other cohorts is needed in future studies. Finally, further prospective clinical studies are needed to verify our findings.

## 5. Conclusions

In conclusion, we systematically analyzed the genetic landscape and biological and clinical relevance of the cGAS-STING signature across human cancers. It helps to better understand the dysregulation of the cGAS-STING pathway in cancers, especially KIRC, KIRP, and PCPG. The findings from the present study can be readily applied to further preclinical translational researches and are highly promising for personalized cancer treatment and clinical application in oncology.

## Data Availability

All data generated or analyzed during this study are included in this article.
